# Pacing during TAVR through a coronary wire in the coronary artery and left ventricular wire

**DOI:** 10.3389/fcvm.2026.1955852

**Published:** 2026-10-22

**Authors:** Ruisong Ma, Lili Zhang, Wang Liao, Sheng Wang

**Affiliations:** 1 Department of Cardiology, Hainan General Hospital, Haikou, China; 2 Hainan Affiliated Hospital, Hainan Medical University, Haikou, China; 3 Hainan Clinical Research Center for Cardiology, Haikou, China

**Keywords:** coronary abstraction, coronary artery wire pacing, J-wire pacing, temporary pacing, transcatheter aortic valve replacement

## Abstract

The conventional pacing-over-the-wire technique in transcatheter aortic valve replacement (TAVR) results in high impedance and voltage thresholds, which may cause loss of capture and valve embolisation. Our team reported that coronary wire pacing, in which a coronary wire is placed in the left coronary artery (anode) and a left ventricular wire (cathode), is associated with extremely low impedance and pacing voltage thresholds in patients who are at high risk of coronary obstruction. Moreover, coronary wire pacing is simple and reliable. In this study, we illustrate the coronary wire pacing technique in a patient to provide a new approach to wire pacing in TAVR.

## Highlights

- Coronary wire pacing in transcatheter aortic valve replacement (TAVR) is simple and reliable.
- Coronary wire pacing in TAVR results in extremely low impedance and pacing voltage thresholds.
- Coronary wire pacing may be a better choice for patients requiring coronary protection

## Introduction

The safety and efficacy of guidewire pacing compared with right ventricular pacing in transcatheter aortic valve replacement (TAVR) have been validated (1). The use of coronary guidewire pacing during coronary angioplasty has also been reported for the last more than 40 years (2). However, whether left ventricular guidewire and coronary guidewire pacing can be applied in TAVR patients with coronary risk who require coronary protection has not been reported to date. In this study, we compared the impedance and pacing thresholds of coronary guidewire pacing, J-guidewire pacing, and conventional guidewire pacing in a patient. Coronary guidewire pacing resulted in lower impedance and pacing thresholds than conventional guidewire pacing and simplified the surgical procedure compared with J-guidewire pacing. Coronary guidewire pacing appears to be a better choice for patients with TAVR who require coronary protection.

## Case presentation

A 78-year-old female patient presented with chest tightness and pain, primarily in the precordial area. Each episode lasted several minutes, which was relieved by rest. The patient denied experiencing syncope, presyncope, or paroxysmal nocturnal dyspnoea. Her outpatient laboratory results indicated a significantly elevated N-terminal pro-brain natriuretic peptide (NT-proBNP) level of 25,271 ng/L (normal range: <450 ng/L), with a cardiac troponin T (cTnT) level of 0.038 μg/L. She has a history of hypertension for more than 30 years, currently managed with valsartan, which has effectively controlled her blood pressure. Upon admission, her physical examination revealed a blood pressure of 159/89 mmHg and scattered moist rales in both lungs. The point of maximal impulse was located 1 cm lateral to the intersection of the left midclavicular line and the sixth intercostal space. A 3/6 systolic blowing murmur was audible in the mitral area, while a 4/6 systolic murmur was audible at the aortic area. No lower-extremity oedema was noted. Laboratory findings included a haemoglobin level of 106 g/L (normal range: 115–150 g/L) and a creatinine level of 72.5 μmol/L (normal range: 41–81 μmol/L). A repeat NT-proBNP level was measured at 10,139 ng/L. The ECG showed no abnormalities. Echocardiography findings revealed an aortic valve peak velocity of 4.24 m/s, with a peak pressure gradient of 72 mmHg and a mean pressure gradient of 43 mmHg. Moderate aortic regurgitation was observed, with a vena contracta width of 3.6 mm, alongside severe mitral regurgitation, which had a vena contracta width of 7.9 mm. The left ventricular diameter was 59 mm (normal range: 0–55 mm), and the ejection fraction was 40% (normal range: 50%–80%).

Aortic Computed Tomography Angiography (CTA): An examination revealed that the aortic valve was trileaflet (tricuspid) with mild calcification (Figure 1A). The mean annular diameter was 22.0 mm (Figure 1B). The left ventricular outflow tract had a diameter of 23.9 mm. The diameters at 2, 4, 6, and 8 mm above the valve measured 21.3, 21.1, 21.4, and 21.8 mm, respectively. The ostial height of the left coronary artery was 6.8 mm, the diameter of the left sinus was 28.2 mm, and the length of the left coronary cusp nearly reached the upper edge of the coronary ostium (Figure 1C). A simulated implantation of a 26 mm valve (Venus Medtech, Hangzhou, China) resulted in a valve frame-to-coronary ostium distance (VTC) of 4.3 mm (Figure 1D). The height of the sinotubular junction was 20.8 mm, and its diameter was 27.7 mm. The ostial height of the right coronary artery was 14.8 mm. The abdominal aorta was tortuous, with multiple calcifications and ulcerated plaques, but no luminal stenosis was observed (Figure 1E).

**Figure 1:**
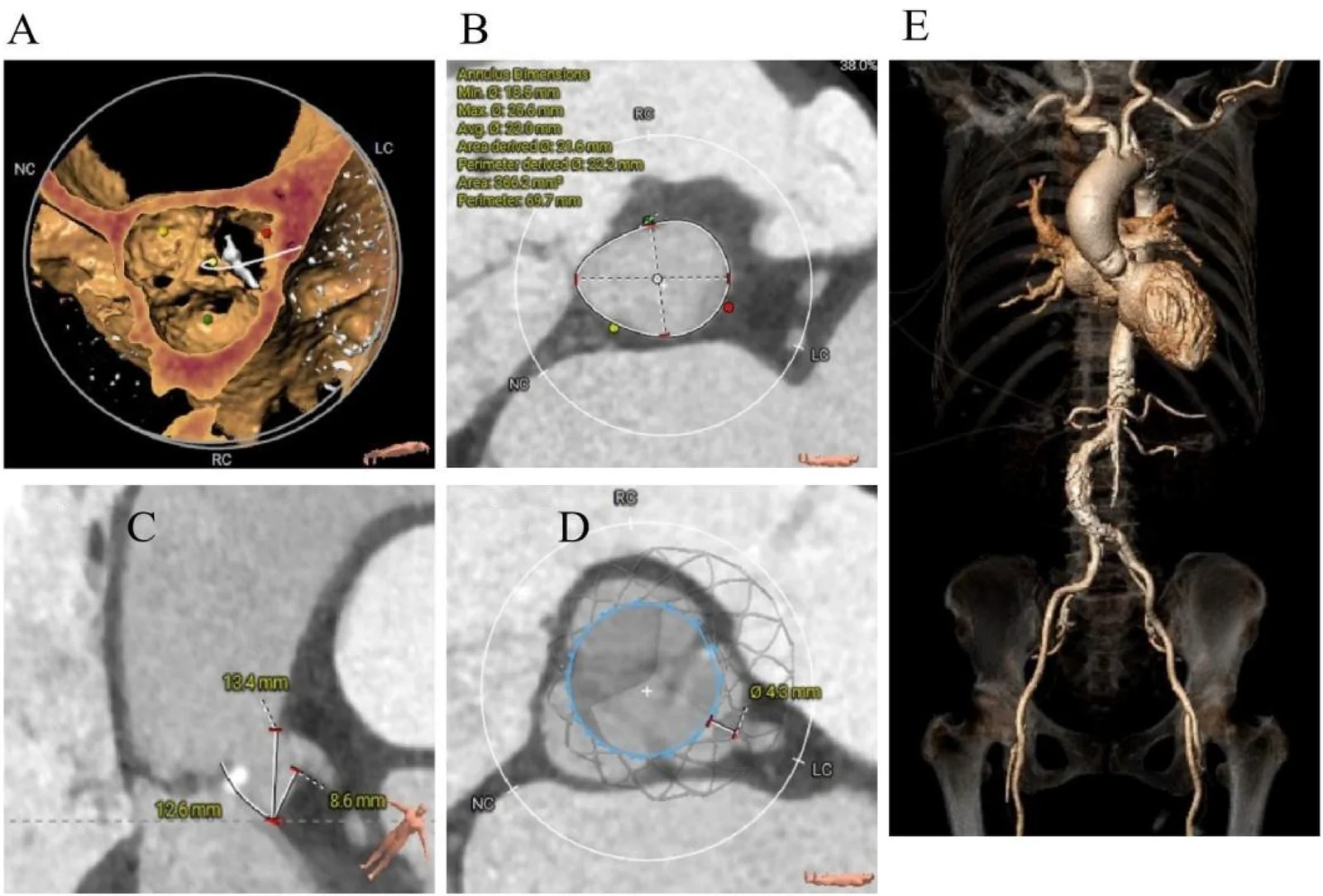
Aortic CTA. **(A)** Tricuspid valve, mild calcification; **(B)** the annular mean diameter is 22.0 mm; **(C)** the left coronary cusp length nearly reaches the upper edge of the coronary ostium; **(D)** simulated implantation of a 26 mm valve gives a VTC 4.3 mm; and **(E)** the abdominal aorta is tortuous with multiple calcifications and ulcerated plaques. VTC, valve frame-to-coronary ostium distance.

Procedural strategy: A 26 mm Venus A valve (Venus Medtech, Hangzhou, China) was used without pre-dilation. The right femoral artery served as the primary access route, with a 45 cm-long sheath advanced to the level of the renal artery. Proactive coronary artery protection was initiated using coronary guidewire pacing.

### TAVR procedure

After anaesthesia, the right femoral artery was punctured, and a 20F femoral artery sheath (APTMedical, Hunan, China) was advanced across the calcified and tortuous segment of the abdominal aorta to the level of the renal artery. The left radial artery was then punctured, and a 6F pigtail catheter was introduced via the left radial artery and positioned at the bottom of the non-coronary sinus for anatomical marking. The right radial artery was punctured, and a 6F EBU guiding catheter (Medtronic, MN, USA) was advanced to the left coronary ostium. An Anyreach C0.6 guidewire (APTMedical, Hunan, China) was advanced to the distal segment of the left anterior descending artery. Along this guidewire, a 6F Guidezilla (Boston Scientific, MA, USA) and a 4.0 mm × 30 mm stent (Medtronic, MN, USA) were advanced to the left main coronary artery for protection. A Lunderquist guidewire (Cook Medical, Inc., Bloomington, IN, USA) was pre-shaped and exchanged into the left ventricle (LV) through the right 20F femoral sheath. To ensure good contact with the LV, the Lunderquist guidewire should show dynamic compression during systole under fluoroscopy. A 26 mm VenusA valve (Venus Medtech, Hangzhou, China) was delivered to the ascending aorta, and different pacing modalities were tested as follows: conventional guidewire pacing (the anode connected to a subcutaneous needle in the groin area or to a grounding pad on the left chest wall), J-wire pacing (the J wire was advanced through the femoral artery sheath to the descending aorta at the same level as the LV guidewire, with the anode connected to the hub end of the J wire), and coronary guidewire pacing (the anode connected to the hub end of the coronary guidewire; Figure 2A). The cathode was connected to the hub end of the Lunderquist guidewire in all configurations (Figure 2B). The results are presented in Table 1. The valve was successfully deployed to the working position under coronary guidewire pacing (10 V) at the standard implantation depth (Supplementary Video S1). During the process of releasing the crown, the valve migrated downward to a deep position. The 6F Guidezilla and coronary stent were withdrawn, and coronary flow was assessed as adequate (Supplementary Video S2). There was trace paravalvular aortic regurgitation, with a transvalvular pressure gradient of 3 mmHg. Therefore, coronary stenting was not performed (Supplementary Video S3).

**Figure 2:**
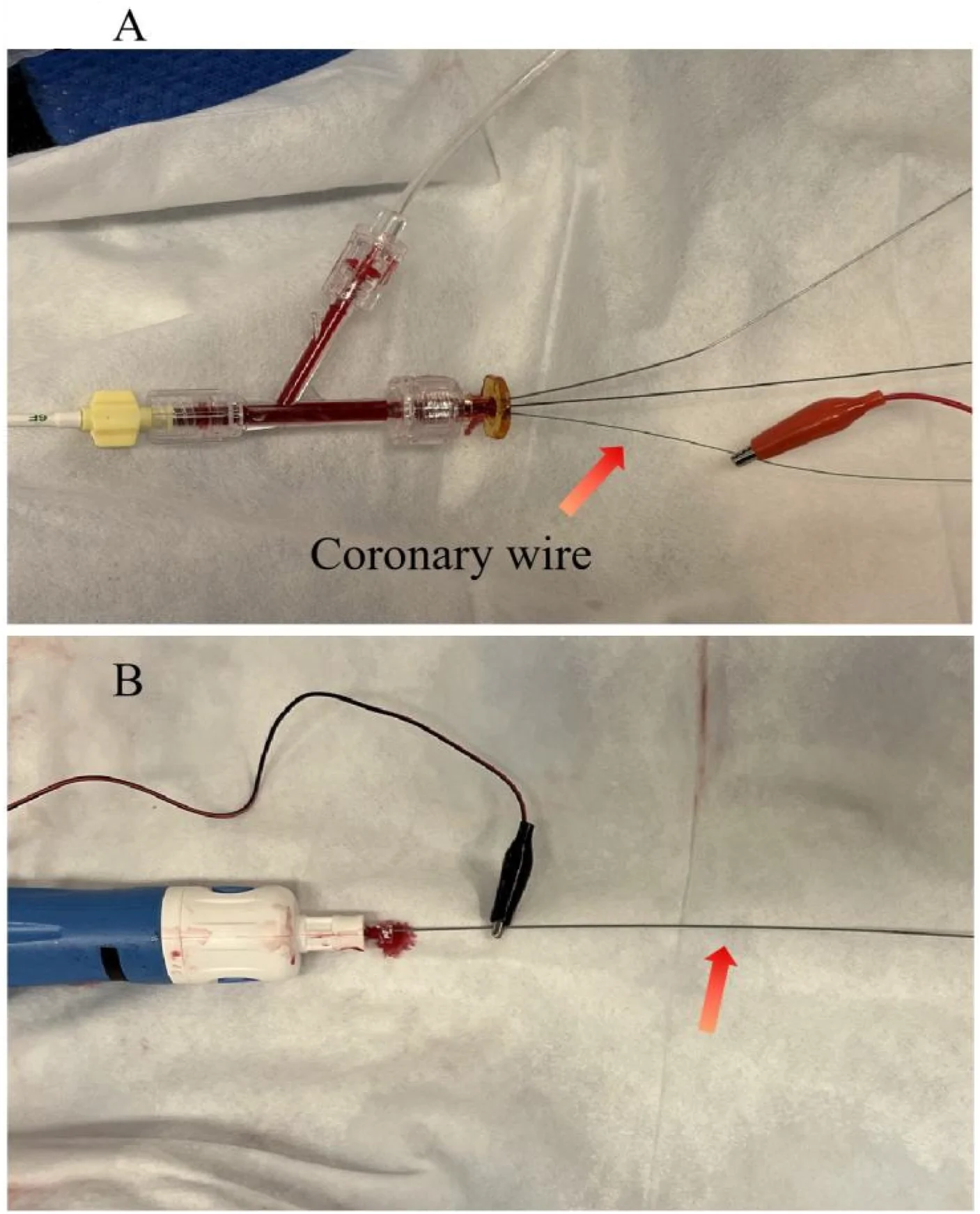
Coronary wire pacing. **(A)** The anode connected to the tip of the coronary guidewire; and **(B)** the cathode connected to the tip of the Lunderquist guidewire.

**Table 1:** Impedance and voltage thresholds in different LV wire pacing modes.

Impedance and voltage thresholds	Grounding pad	Subcutaneous needle	J wire	Coronary wire
Voltage (V)	10	8	4	4
Impedance (Ω)	850	500	220	220

The impedance and voltage thresholds of four different wire pacing models were detected, namely, the grounding pad, the subcutaneous needle, J-wire pacing, and coronary wire pacing.

## Discussion

In the conventional pacing-over-the-wire technique, a grounding pad placed at the apex or a needle inserted into the subcutaneous tissue is used. The greatest risk associated with this method is the high pacing voltage threshold, which can lead to a loss of LV capture rate, occurring in 0.5%–4% of cases. To reduce the risk of LV loss of capture, the pacing voltage needs to be set above twice the voltage threshold, which limits the widespread application of LV wire pacing (3). It is not uncommon for patients undergoing TAVR to require coronary protection. In this subset of patients, the use of coronary guidewire pacing not only reduces the risk of local tissue injury and muscle twitching associated with conventional pacing wires but also significantly lowers pacing thresholds and impedance, thereby reducing the risk of loss of capture and improving procedural safety. The application of coronary guidewire pacing in coronary angioplasty has been established for more than 40 years (2), and its safety has been validated. Therefore, in patients requiring coronary protection, coronary guidewire pacing may be a safer and more effective option compared with conventional guidewire pacing.

Our team previously proposed J-wire pacing, which involves delivering a J wire to the descending aorta via a 20F femoral sheath (for self-expanding valves) or an 8F accessory femoral sheath (for balloon-expandable valves) at a height aligned with the left ventricular guidewire (4). Compared with conventional guidewire pacing, J-wire pacing significantly reduces pacing thresholds and impedance but may mildly increase intraoperative bleeding. In patients with TAVR requiring coronary protection, coronary guidewire pacing not only simplifies the procedure and avoids additional intraoperative blood loss compared with J-wire pacing, but also, more importantly, provides pacing thresholds and impedance comparable to those of J-wire pacing. Therefore, in patients who need coronary protection, coronary guidewire pacing appears to be a superior choice compared with J-wire pacing.

In conclusion, in patients requiring coronary protection, coronary guidewire pacing offers a low threshold and impedance, demonstrates good safety and efficacy, and simplifies the procedure, making it a superior choice.

### Limitations

Coronary guidewire pacing is used only for short-term intraoperative emergency pacing, and its application has the following limitations: (1) it is used only in patients who develop high coronary risk, as its use in other patients would require additional arterial access and increase costs; (2) it is not suitable for patients who require prolonged pacing during the procedure. For example, the pacing demand is expected to exceed the brief window of balloon dilation or valve deployment, or when continuous conduction monitoring is required postoperatively; (3) it is not suitable for patients at high risk of conduction block, such as those with pre-existing right bundle branch block; and (4) it may increase the risk of coronary injury and perforation, especially when the coronary guidewire is poorly positioned.

## Data Availability

The original contributions presented in the study are included in the article/Supplementary Material, further inquiries can be directed to the corresponding author.

## Supplementary material

The Supplementary Material for this article can be found online at: https://www.frontiersin.org/articles/10.3389/fcvm.2026.1955852/full#supplementary-material

SUPPLEMENTARY VIDEO S1 The valve was successfully deployed to the working position under coronary guidewire pacing at the standard implantation depth.

SUPPLEMENTARY VIDEO S2 The 6F Guidezilla and the coronary stent were withdrawn to the outer aspect of the left main artery, and left coronary artery flow was confirmed to be unaffected.

SUPPLEMENTARY VIDEO S3 The coronary guidewire was withdrawn, and left coronary artery flow was again confirmed to be unaffected.
